# Observational and Genetic Evidence for Bidirectional Effects Between Red Blood Cell Traits and Diastolic Blood Pressure

**DOI:** 10.1093/ajh/hpad061

**Published:** 2023-07-11

**Authors:** Zhen He, Zekai Chen, Martin H de Borst, Qingying Zhang, International Consortium of Blood Pressure, Harold Snieder, Chris H L Thio

**Affiliations:** Department of Epidemiology, University of Groningen, University Medical Center Groningen, Groningen, The Netherlands; Department of Preventive Medicine, Shantou University Medical College, Shantou, Guangdong, PR China; Department of Epidemiology, University of Groningen, University Medical Center Groningen, Groningen, The Netherlands; Department of Internal Medicine, Division of Nephrology, University of Groningen, University Medical Center Groningen, Groningen, The Netherlands; Department of Preventive Medicine, Shantou University Medical College, Shantou, Guangdong, PR China; Department of Epidemiology, University of Groningen, University Medical Center Groningen, Groningen, The Netherlands; Department of Epidemiology, University of Groningen, University Medical Center Groningen, Groningen, The Netherlands

**Keywords:** blood pressure, hemoglobin, hypertension, Mendelian randomization, red blood cell count

## Abstract

**BACKGROUND:**

Previous studies have found associations of red blood cell (RBC) traits (hemoglobin and RBC count) with blood pressure; whether these associations are causal is unknown.

**METHODS:**

We performed cross-sectional analyses in the Lifelines Cohort Study (*n* = 167,785). Additionally, we performed bidirectional 2 sample Mendelian randomization (MR) analyses to explore the causal effect of the 2 traits on systolic (SBP) and diastolic blood pressure (DBP), using genetic instrumental variables regarding hemoglobin and RBC identified in UK Biobank (*n* = 350,475) and International Consortium of Blood Pressure studies for SBP and DBP (*n* = 757,601).

**RESULTS:**

In cross-sectional analyses, we observed positive associations with hypertension and blood pressure for both hemoglobin (odds ratio [OR] = 1.18, 95% confidence interval [CI]: 1.16–1.20 for hypertension; *B* = 0.11, 95% CI: 0.11–0.12 for SBP; *B* = 0.11, 95% CI: 0.10–0.11 for DBP, all per SD) and RBC (OR = 1.14, 95% CI: 1.12–1.16 for hypertension; *B* = 0.11, 95% CI: 0.10–0.12 for SBP; *B* = 0.08, 95% CI: 0.08–0.09 for DBP, all per SD). MR analyses suggested that higher hemoglobin and RBC cause higher DBP (inverse-variance weighted *B* = 0.11, 95% CI: 0.07–0.16 for hemoglobin; *B* = 0.07, 95% CI: 0.04–0.10 for RBC, all per SD). Reverse MR analyses (all per SD) suggested causal effects of DBP on both hemoglobin (*B* = 0.06, 95% CI: 0.03–0.09) and RBC (*B* = 0.08, 95% CI: 0.04–0.11). No significant effects on SBP were found.

**CONCLUSIONS:**

Our results suggest bidirectional causal relationships of hemoglobin and RBC with DBP, but not with SBP.

## Graphical abstract

**Graphical Abstract:**
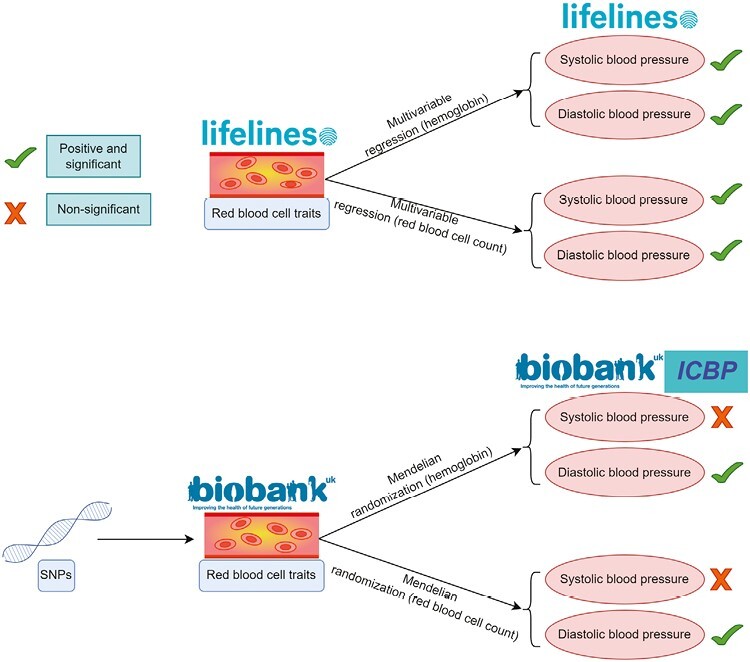

Hypertension is an important cause of cardiovascular disease, premature death, chronic kidney disease, and dementia.^1,2^ Globally, hypertension prevalence continues to rise. In 2015, the number of deaths related to high blood pressure was more than 8 million, and the vast majority (88%) of them occurred in low- and middle-income countries.^2^ Modifiable risk factors for hypertension include low potassium intake, high sodium intake, overweight and obesity, unhealthy diet, lack of physical activity, cigarette smoking, and alcohol consumption.^1^ The pathogenesis underlying hypertension is complex and incompletely understood; further study into potential mechanisms is therefore warranted.

Hemoglobin, the principal component of red blood cells (RBCs), performs an important function transporting oxygen and carbon dioxide into and out of the body’s circulation system.^3^ Red blood cells are a primary determinant of blood viscosity, which has been suggested to be linked to blood pressure^4^ and cardiovascular events.^5^ Previous observational studies in different populations support such a link,^6–12^ whereas in trials in clinical samples (chronic kidney disease, diabetes, and anemia), erythropoietin/erythropoiesis-stimulating agents caused elevated blood pressure.^13,14^ However, the observational studies are possibly affected by unmeasured confounding and reverse causation. Furthermore, generalizability of results from trials in clinical samples to the general population is uncertain. Thus, it remains unknown whether hemoglobin and RBC are causally related with blood pressure in the general population.

Under the instrumental variable (IV) assumptions, the Mendelian randomization (MR) approach can overcome some of the problems of observational studies, such as confounding and reverse causation, thereby strengthening causal inference from observational data.^15^

In the current study, we aimed to examine potential effects of 2 RBC traits (i.e., hemoglobin and RBC) on blood pressure by applying 2 approaches within a triangulation framework^16^ (integrating results from different approaches with different key sources of bias to strengthen confidence in the findings) (Figure 1): first, multivariable-adjusted observational analysis in cross-sectional data from Lifelines, a large prospective cohort involving more than 167,000 inhabitants of the northern part of the Netherlands,^17^ and second, 2-sample MR analysis using publicly available genome-wide association study (GWAS) summary data.^18,19^

**Figure 1.**
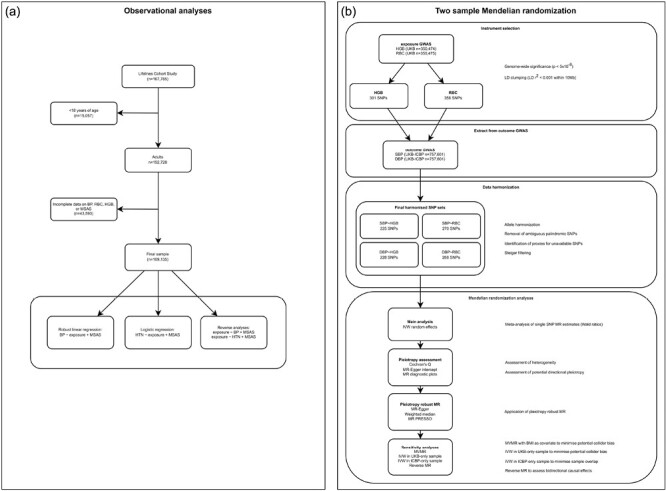
Flowchart of observational (Panel a) and 2-sample Mendelian randomization analyses (Panel b). Abbreviations: BMI, body mass index; BP, blood pressure; DBP, diastolic blood pressure; eGFR, estimated glomerular filtration rate; HGB, hemoglobin; HTN, hypertension; IVW, inverse-variance weighted; LD, linkage disequilibrium; MSAS, minimal sufficient adjustment sets; MSAS included age, gender, BMI, education, physical exercise, eGFR, and smoking; MR, Mendelian randomization; RBC, red blood cell count; SBP, systolic blood pressure; SNPs, single-nucleotide polymorphisms.

## METHODS

### Observational analysis in the Lifelines Cohort Study

#### Study population

The overall design of the Lifelines Cohort Study has previously been described in detail elsewhere.^17^ In brief, Lifelines is a multidisciplinary prospective population-based cohort study examining in a unique 3-generation design the health and health-related behaviors of 167,785 persons living in the North of the Netherlands. It employs a broad range of investigative procedures in assessing the biomedical, sociodemographic, behavioral, physical, and psychological factors which contribute to the health and disease of the general population, with a special focus on multimorbidity and complex genetics. The study adhered to the Declaration of Helsinki and was approved by the medical ethical committee (number 2007/152) of the University Medical Center Groningen, The Netherlands. Signed informed consent statements were obtained for all study participants prior to inclusion to the study. We created a directed acyclic graph (DAG) (Supplementary Figure S1 online) using DAGitty v3.0 (http://dagitty.net/dags.html)^20^ to identify a minimally sufficient adjustment set (MSAS). This MSAS included age, sex, body mass index (BMI; weight divided by height^2^), education (no college degree; college degree or higher), nonoccupational physical activity (moderate-to-vigorous-intensity minutes of participants in physical activity per week in leisure time and commuting domains [but not occupational]), estimated glomerular filtration rate (estimated by CKD-EPI equation^21^), and smoking (never smoker, ex-smoker, current smoker). We sampled adult subjects (≥18 years of age) from the Lifelines Cohort Study that had complete data on blood pressure (mean of the last 3 measurements using an automatic blood pressure monitor [DinaMap, PRO 100V2]^22^), hemoglobin, RBC (determined by a Sysmex XE-2100 analyzer [Sysmex Corporation, Kobe, Japan]), and the variables in the MSAS. Hypertension was defined as systolic blood pressure (SBP) ≥140 mm Hg and/or diastolic blood pressure (DBP) ≥90 mm Hg or use of antihypertensive medication.^23^ In addition, we performed analysis using hypertension defined as SBP ≥130 mm Hg and/or DBP ≥80 mm Hg, as per the updated guidelines of the American College of Cardiology/American Heart Association (ACC/AHA).^24^ For those participants taking antihypertensive agents, SBP and DBP were adjusted by adding 15 and 10 mm Hg, respectively, to their observed values, which is a recommended approach to reduce bias and improve statistical power.^25^ Subjects (*n* = 43,593) with missing data on hemoglobin, RBC, blood pressure and any of the covariates from the MSAS were removed, leaving 109,135 participants for the analysis (Figure 1).

#### Statistical analysis

Continuous data were represented as mean ± SD or median (interquartile range [IQR]), while categorical data were expressed by numbers with percentages. For each outcome (i.e., hypertension, SBP, and DBP), we constructed 3 models: model 1 was an unadjusted model; model 2 was adjusted for age and sex; model 3 was adjusted for the MSAS. Reverse analyses were performed using BP or hypertension as exposure, and hemoglobin and RBC as outcomes, while adjusting for the same set of variables. We applied a robust linear regression method as this method, compared with ordinary linear regression, is considered less sensitive to the wide distribution of blood pressure, especially potential outliers therein, we observed in our data (see Supplementary Figure S2 online). The *stats* and *robustbase* packages in R (version 4.0.3) were used. A 2-sided *P* < 0.0125 (0.05/4, based on a Bonferroni correction for 4 tests) was considered statistically significant. We standardized our traits of interest (i.e., hemoglobin, RBC, SBP, and DBP) so that each had a mean of 0 and a variance of 1.

Reporting of the present cross-sectional study conformed to the STROBE Cross-sectional study Statement^26^ (Supplementary Table S1 online).

### MR analysis

#### Data sources

We used UK Biobank (UKB, *n* = 458,577 Europeans) and International Consortium of Blood Pressure-GWAS (ICBP, *n* = 299,024 Europeans, from 77 different cohorts)^18,19^ in this MR study. Details on these studies can be found in Supplementary Table S2 online.

#### MR rationale

Due to Mendel’s laws of random segregation and independent assortment, genetic variants for an exposure are unlikely to be related to confounders. Furthermore, formation of zygotes precedes any exposures and outcomes. Thus, genetic variants mimic a natural experiment and can therefore be exploited as IVs in MR analysis. MR yields unbiased causal estimates under 3 key assumptions: relevance, exchangeability, and exclusion restriction.^27^

#### Exposure data

Blood samples were collected in 4 ml EDTA vacutainers for participants of the UK Biobank baseline cohort and analyzed at the UK Biobank central laboratory within 24 hours of blood draw. Measurement of hemoglobin and RBC was determined by Beckman Coulter LH750 Haematology Analyser (Beckman Coulter, Fullerton, California, USA).^18^

#### Instrument selection

From UKB GWAS summary data, we selected single-nucleotide polymorphisms (SNPs) according to the standard process (Figure 1).^28^ Instrument strength was assessed by calculating *F*-statistics.^29^ Details of the IV selection are provided in Figure 1, with SNP details presented in Supplementary Tables S3 and S4 online.

#### Outcome data

We obtained summary data for the association of the hemoglobin and RBC-related variants with blood pressure from combined GWAS studies (*n* = 757,601 Europeans, presented as “UKB-ICBP”), which is composed of UKB (*n* = 458,577 Europeans) and the ICBP-GWAS (*n* = 299,024 Europeans, from 77 different cohorts).^18,19^ For ease of comparison, we standardized blood pressure summary GWAS data using a *Z*-score transformation.

#### Statistical analysis

Random-effects inverse-variance weighted (IVW) meta-analysis of the Wald ratio (i.e., effect of SNP on outcome divided by effect of SNP on exposure^27^) for each included SNP was performed, which is a common approach to estimate the causal effect under the assumption that all SNPs are valid IVs.^28^ Several sensitivity analyses were conducted, i.e., heterogeneity assessment, leave-one-out MR analysis, outlier assessment, Mendelian Randomization-Egger (MR-Egger) regression,^30^ weighted median method,^31^ MR Pleiotropy RESidual Sum and Outlier (MR-PRESSO),^32^ and Steiger filtering.^33^ We assessed heterogeneity by Cochran’s *Q*-statistic. We examined individual Wald estimates, leave-one-out analyses, and funnel plots to identify influential, potentially pleiotropic SNPs and overall directional pleiotropy.

Because the results before and after Steiger filtering gave highly consistent estimates, we only report the results (except for multivariable MR [MVMR]) after Steiger filtering. To further investigate potential reverse causation, reverse MR analyses (i.e., using SBP/DBP as exposures and hemoglobin/RBC as outcomes) were performed using genetic instruments for SBP and DBP.

The UKB-ICBP data were adjusted for BMI, a potential intermediate in the SNP–BP relation. Therefore, collider bias may have been introduced.^20^ We therefore performed sensitivity analyses using summary data on blood pressure from UKB (presented as “UKB only”) that were not adjusted for BMI. However, this resulted in complete sample overlap which may exacerbate weak instrument bias and bias toward the observational estimate.^15^ We therefore performed additional sensitivity analyses using summary data from ICBP (presented as “ICBP only,” adjusted for BMI). In addition, we applied MVMR including BMI as a covariate as this has recently been shown to counter potential collider bias due to BMI adjustment.^34^ Here, we did not apply Steiger filtering,^33^ as it is not well established in the MVMR framework. Due to overlap of SNPs between hemoglobin and RBC, as well as between SBP and DBP, there is potential violation of the exclusion restriction assumption through horizontal pleiotropy (Supplementary Figure S3 online). To address this issue, we additionally conducted MVMR analysis in which we simultaneously modeled the effects of hemoglobin and RBC count on blood pressure, as well as in reverse analysis in which we simultaneously modeled effects of SBP and DBP on RBC traits. To further investigate the robustness of our DBP findings, we conducted a phenome-wide association study (PheWAS) by querying the IEU OpenGWAS repository (https://gwas.mrcieu.ac.uk/)^35^ to identify potential pleiotropic variants genome-wide significantly (*P* < 5 × 10^−8^) associated with any of the *a priori* defined confounders (see Supplementary Figure S1 online). We then performed sensitivity analysis in which we removed these SNPs. Finally, we performed exploratory MR analysis on RBC distribution width, mean corpuscular volume, mean corpuscular hemoglobin concentration, mean corpuscular hemoglobin, and hematocrit and blood pressure (more details on methods in Supplementary Figure S15 online).

MR results were visualized using forest plots and scatter plots, funnel plots, and leave-one-out plots. *TwoSampleMR*, *ieugwasr*, and *MRPRESSO* packages in R (versions 3.6.2 and 4.0.3) were used to perform this MR analysis. A 2-sided threshold of *P* < 0.0125 (0.05/4, based on the Bonferroni correction) was considered statistically significant. This MR study followed the STROBE-MR Checklist of Recommended Items to Address in Reports of MR Studies (Supplementary Table S5 online).^36^

## RESULTS

### Observational results in the Lifelines Cohort Study

In total, 109,135 subjects with complete data were enrolled into the current Lifelines cross-sectional study. The median age was 45 (IQR 37–53) years. Mean ± SD of hemoglobin and RBC were 14.11 ± 1.27 g/dl and 4.71 ± 0.40 (×10^12^)/l, respectively. Medians of SBP and DBP were 125 (IQR 115–137) mm Hg and 74 (IQR 68–81) mm Hg, respectively. Prevalence of hypertension in this population was 26.1% (28,484/109,135) (Supplementary Table S6 online). Similar characteristics were observed in adult data containing the complete cases (*n* = 152,728) (Supplementary Table S6 online), and the possibility of selection bias was low. We observed a similar positive association of blood pressure (including SBP and DBP) to both hemoglobin and RBC in boxplots (Supplementary Figure S2 online).

**Figure 2.**
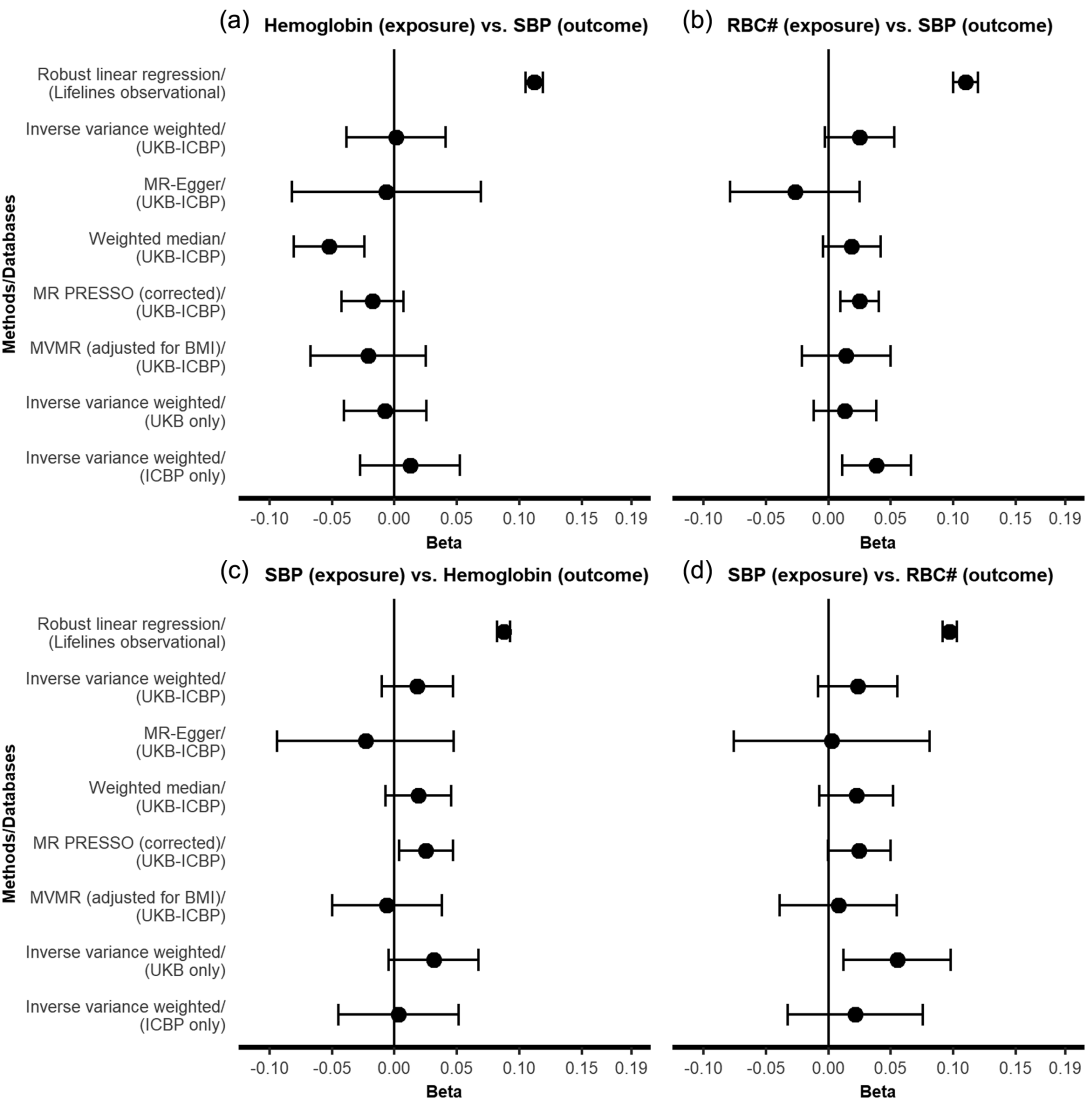
Comparison of bidirectional effect estimates between observational, Mendelian randomization, and Mendelian randomization sensitivity analyses for systolic blood pressure. Panels (a) and (b) show the results of forward analyses with hemoglobin and red blood cell count as the exposures and SBP as the outcome. Panels (c) and (d) show the results of reverse analyses with SBP as the exposure and hemoglobin and red blood cell count as the outcomes. The *X*-axis indicates effect size as SDs difference in outcome per 1 SD higher value of exposure. The *Y*-axis indicates analysis method (with their respective data source). Error bars indicate 95% confidence interval (CI). Lifelines observational regression estimates were adjusted for age, sex, BMI, education, nonoccupational physical activity, eGFR, and smoking. Abbreviations: BMI, body mass index; eGFR, estimated glomerular filtration rate; ICBP, International Consortium of Blood Pressure; MR-PRESSO, Mendelian Randomization Pleiotropy RESidual Sum and Outlier; MVMR, multivariable Mendelian randomization; RBC, red blood cell count; SBP, systolic blood pressure; UKB, UK Biobank.

**Figure 3.**
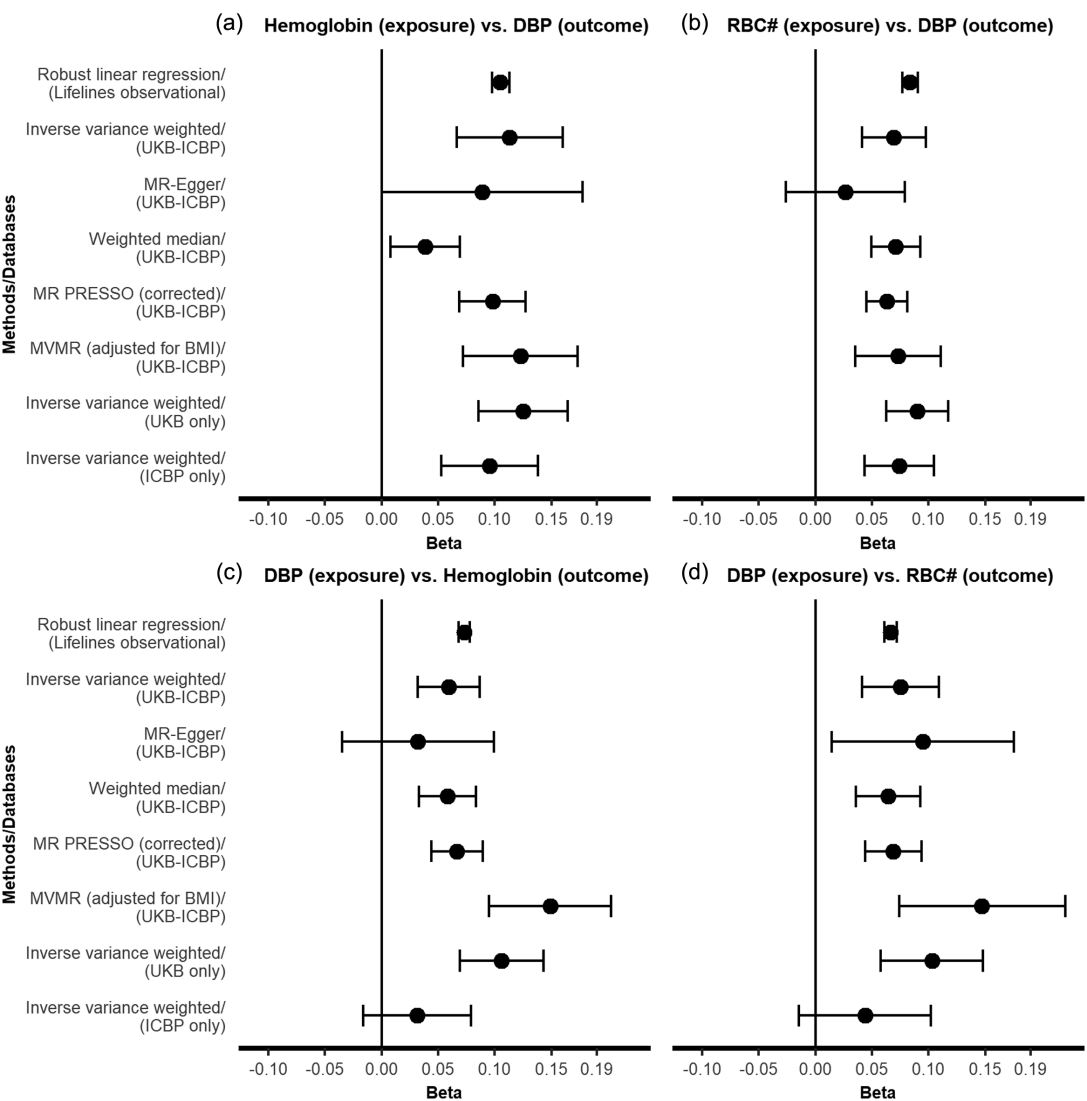
Comparison of bidirectional effect estimates between observational, Mendelian randomization, and Mendelian randomization sensitivity analyses for diastolic blood pressure. Panels (a) and (b) show the results of forward analyses with hemoglobin and red blood cell count as the exposures and DBP as the outcome. Panels (c) and (d) show the results of reverse analyses with DBP as the exposure and hemoglobin and red blood cell count as the outcomes. The *X*-axis indicates effect size as SDs difference in outcome per 1 SD higher value of exposure. The *Y*-axis indicates analysis method (with their respective data source). Error bars indicate 95% confidence interval (CI). Lifelines observational regression estimates were adjusted for age, sex, BMI, education, nonoccupational physical activity, eGFR, and smoking. Abbreviations: BMI, body mass index; DBP, diastolic blood pressure; eGFR, estimated glomerular filtration rate; ICBP, International Consortium of Blood Pressure; MR-PRESSO, Mendelian Randomization Pleiotropy RESidual Sum and Outlier; MVMR, multivariable Mendelian randomization; RBC, red blood cell count; UKB, UK Biobank.

A total of 109,135 subjects with complete information were included in the multivariable analyses. Hemoglobin was positively associated with hypertension (odds ratio [OR] = 1.18 per SD higher hemoglobin, 95% confidence interval [CI]: 1.16–1.20), SBP (*B* = 0.11 SD per SD higher hemoglobin, 95% CI: 0.11–0.12), and DBP (*B* = 0.11 SD per SD higher hemoglobin, 95% CI: 0.10–0.11) in the MSAS-adjusted models. Furthermore, RBC was also positively associated with hypertension (OR = 1.14 per SD higher RBC, 95% CI: 1.12–1.16), SBP (*B* = 0.11 SD per SD higher RBC, 95% CI: 0.10–0.12), and DBP (*B* = 0.08 SD per SD higher RBC, 95% CI: 0.08–0.09) in the MSAS-adjusted models (Figures 2 and 3, Supplementary Table S7 online).

Inverse estimates (i.e., hypertension/SBP/DBP as exposures and hemoglobin/RBC as outcomes) are shown in Figures 2 and3, Supplementary Table S8 online. Analyses on hypertension defined by ACC/AHA guidelines (i.e., 130/80 mm Hg) yielded larger effect sizes compared with the previous criteria of 140/90 mm Hg (Supplementary Tables S7 and S8 online).

### MR results

We calculated a 1-sided lower-bound confidence limit of the *F* parameter for the 2 traits and found sufficient instrument strength (91.1 for hemoglobin and 107.1 for RBC). All individual SNPs had an *F* > 10, which is further evidence of sufficient instrument strength.

We found no significant effects of hemoglobin on SBP (IVW *B* = 0.00 SD per SD higher hemoglobin, 95% CI: −0.04 to 0.04) or RBC on SBP (IVW *B* = 0.03 SD per SD higher hemoglobin, 95% CI: −0.00 to 0.05) (Figure 2a,b, Supplementary Table S9 online). On the other hand, there were positive associations of hemoglobin on DBP (IVW *B* = 0.11 SD per SD higher hemoglobin, 95% CI: 0.07–0.16) and RBC on DBP (IVW *B* = 0.07 SD per SD higher hemoglobin, 95% CI: 0.04–0.10). MR sensitivity analyses yielded consistent results (Figure 3a,b, Supplementary Table S9 online). These DBP findings translate to mm Hg as follows: hemoglobin was positively associated with DBP (*B* = 1.03 mm Hg per SD hemoglobin, 95% CI: 0.95–1.10 for observational analysis; IVW *B* = 1.21 mm Hg per SD hemoglobin, 95% CI: 0.71–1.71 for MR analysis). RBC was positively associated with DBP (*B* = 0.80 mm Hg per SD RBC, 95% CI: 0.73–0.87 for observational analysis; IVW *B* = 0.74 mm Hg per SD RBC, 95% CI: 0.44–1.04 for MR analysis).

There was no evidence of directional pleiotropy for the hemoglobin–DBP (MR-Egger intercept *P* value = 0.527) and the RBC–DBP analysis (MR-Egger intercept *P* value = 0.059). The estimates of MVMR and IVW models from UKB-ICBP, UKB only and ICBP only datasets were again consistent, suggesting that our results were unlikely to be severely affected by collider bias (Figures 2 and 3, Supplementary Table S9 online). We conducted MVMR analysis to account for potential horizontal pleiotropy. MVMR results corroborated our findings of RBC traits on DBP, although there was attenuation of the effect of RBC count on DBP, suggesting that either (i) effects on DBP are primarily driven by hemoglobin, or (ii) effects of RBC count on DBP are mediated through hemoglobin (Supplementary Table S9 online). Interestingly, reverse analysis showed stronger effects of DBP on RBC traits. PheWAS identified several potential pleiotropic SNPs (Supplementary Table S10 online). Removal of these SNPs in sensitivity MR analyses yielded consistent results (Supplementary Table S9 online). Very similar patterns were observed in the reverse MR analyses and its sensitivity analyses using blood pressure as exposure, suggesting an effect of DBP (but not SBP) on hemoglobin and RBC (Figures 2 and 3, Supplementary Figures S4 and S5 online). Single SNP forest plots, funnel plots, and leave-one-out plots were indicative of heterogeneity, but were not suggestive of directional bias due to influential, potentially pleiotropic SNPs (Supplementary Figures S6–S13 online). Exploratory MR and observational analyses show a positive association of hematocrit with DBP inconsistent associations with SBP. No associations for other RBC traits (i.e., RBC distribution width, mean corpuscular volume, mean corpuscular hemoglobin concentration, mean corpuscular hemoglobin) and blood pressure were observed in MR analysis (Supplementary Figures S14 and S15, Tables S9 and S11 online).

## DISCUSSION

The present study used complementary observational and MR analyses to investigate the causal relationship of hemoglobin and RBC with blood pressure. We found converging evidence for a bidirectional causal effect between hemoglobin, RBC, and DBP, and a null effect in either direction for SBP.

Our observational results are consistent with previous studies.^6–8,13,37^ Atsma *et al.* revealed that SBP was 1.8 mm Hg higher (95% CI: 1.6–2.0) and 1.3 mm Hg (95% CI: 1.1–1.4) per mmol/l higher hemoglobin for women and men, respectively, based on a large sample of more than 100,000 Dutch voluntary blood donors, and similar results were found for DBP (*B*: 1.5, 95% CI: 1.4–1.6 for women; *B*: 1.4, 95% CI: 1.3–1.5 for men),^6^ which supports our findings. Interestingly, a large randomized, double-blind, placebo-controlled trial (*n* = 4,038 patients with chronic kidney disease, diabetes, and anemia) showed an increase in DBP (73 mm Hg [IQR 67–78] vs. 71mm Hg [IQR 65–77]; *P* < 0.001) after administration of darbepoetin alfa, an erythropoiesis-stimulating agent. Concordant with our study, SBP did not increase.^13^ In an open-label trial involving 1,432 patients with chronic kidney disease in the United States, DBP increased by 0.2 ± 12.9 mm Hg in high-hemoglobin group and decreased by 0.7 ± 12.4 mm Hg in low-hemoglobin group, whereas SBP decreased in the both groups compared with baseline levels, although the impact of antihypertensive drugs cannot be ruled out.^14^ Discordant with our study, a large MR study (*n* = 678,320) using a strong functionally validated instrument suggested that higher endogenous erythropoietin levels causes lower DBP (*B*: −0.98, 95% CI: −1.67 to −0.29), but not lower SBP (*B*: 0.53, 95% CI: −0.65 to 1.71).^38^ A potential explanation for this discordant effect is that endogenous erythropoietin levels may also be higher due to potential compensatory mechanisms in response to proneness to anemia; endogenous erythropoietin, as an upstream proxy of RBC and hemoglobin, may thus potentially show opposite effects to these traits. In a 24-week, multicenter trial from 15 countries (*n* = 252 chronic kidney disease patients), daprodustat (an oral hypoxia-inducible factor-prolyl hydroxylase inhibitor) and controls (received recombinant human erythropoietin) did not markedly impact SBP and DBP. The authors propose that this was related to smaller drug doses and/or adequate control of blood pressure.^39^ None of the abovementioned studies examined a potential reverse causal relation between hemoglobin/RBC and DBP. In summary, our results are generally consistent with previous studies with large sample size and suggest that hemoglobin and RBC contribute to a higher DBP and the development of hypertension, although some inconsistencies remain that warrant follow-up study.

We found evidence for causal effects of hemoglobin/RBC on DBP. A possible explanation is that microparticles released from RBCs could exert its function in inactivation of nitric oxide (NO),^40^ which has an antihypertensive effect.^41^ Not only that, arginase released by hemolysis can retard NO generation.^40^ Moreover, free hemoglobin, even when present at low levels, released by damaged RBCs could not only play a role in scavenging NO activity through dioxygenation reactions, but also induce vasoconstriction, inflammation, and oxidative stress, a state with greater amount of reactive oxygen species than the normal physiological range.^40,42,43^ The generation of these reactive oxygen species might also suppress the bioactivity of NO.^44^ In our study, we only found associations of hemoglobin and RBC with DBP, but not with SBP. One possible explanation is that the abovementioned microparticles, arginase and free hemoglobin from RBCs have the function of scavenging NO activity.^40^ While blood flow velocity around the center of the vessel is higher than that near the walls of blood vessels attributed to the friction force. This forms a pressure difference that drives RBCs toward the center of the vessel far away from the NO synthesizing endothelium.^40^ Blood flow velocity is not constant in common carotid artery, it is faster during systole than that during diastole.^45^ When the flow velocity is decreased (corresponding to DBP), the endothelium may be more susceptible to exposure to RBCs and its related substances, such as free hemoglobin, compared with the higher flow velocity situation during SBP, thus inactivating NO and inducing higher DBP. In MR, we found a reverse effect of blood pressure on RBC traits, which we did not anticipate. We are unaware of previous literature describing such an effect, and more research is needed to validate this finding and elucidate the mechanisms (if any) underlying this relation. In exploratory analyses, we in addition found evidence for a bidirectional relation between hematocrit and DBP. We found no evidence for a relation of RBC distribution width, mean corpuscular volume, mean corpuscular hemoglobin concentration, or mean corpuscular hemoglobin, with blood pressure. This supports a role for RBC count and total hemoglobin, rather than RBC volume or corpuscular hemoglobin. Furthermore, it lends support for previous observations that polycythemia is (potentially bidirectionally) related to hypertension,^46–48^ given that even with physiologically higher RBC counts and hemoglobin concentrations, effects on blood pressure are present.

MR studies provide unconfounded estimates for an exposure–outcome pair under the IV assumptions discussed in the Methods section. Arguably, horizontal pleiotropy of instrumental SNPs, which is a violation of the exclusion restriction criterion,^15^ is the greatest threat to MR. We therefore performed several sensitivity analyses that allow for a range of violations of this criterion. These analyses yielded consistent results and therefore we deem risk of bias due to horizontal pleiotropy small.

This is the largest study to date that investigated the associations between the RBC traits, hemoglobin and RBC, and blood pressure. Our observational analysis utilizes extensive information on covariates, allowing us to explore the optimal set of confounders. An additional advantage of our analyses is that the complementary design (by comparing observational and MR analyses) increases the reliability of these findings.

Our study also has some limitations. First, high-level evidence to inform the DAG, such as prospective and experimental studies, was not always available. This may have led to misoriented arrows in the DAG, and as a result, suboptimal covariate adjustment. Second, at the time of analysis, we only had data on medication use for baseline and not for follow-up; this precluded meaningful longitudinal observational analyses. Third, MR is thought to estimate a lifetime effect of an exposure on an outcome; short-term effects or potential treatment effects may be smaller. Fourth, our study was done in samples of the general population of European ancestry; generalization to the clinic and other ethnicities is therefore uncertain. Fifth, in MR, associations can be interpreted as causal effects, but only if the MR assumptions (some of which untestable) hold. Functional work is required to definitively establish causality.

Our findings suggest a modest causal effect of RBC traits on DBP, providing insights into physiology. Future study could assess whether intervention on RBC traits would translate into meaningful protection against hypertension and sequelae thereof, and whether it is safe to do so given the risk of anemia. If not suitable as a drug target, RBC traits could instead play a role in risk stratification; future study could assess whether inclusion of RBC traits, on top of traditional risk factors, improves prediction of hypertension. Additionally, future work may involve investigating potential sex differences in the relation between hemoglobin and RBC with blood pressure, using sex-stratified GWAS data.

In conclusion, our results suggest bidirectional causal relationships between hemoglobin, RBC, and DBP, but no causal relationships with SBP.

## Supplementary Material

hpad061_suppl_Supplementary_Material

hpad061_suppl_Supplementary_Tables

## Data Availability

The Lifelines cohort data can be applied through the link: http://wiki-lifelines.web.rug.nl/doku.php?id=start, and UKB and UKB-ICBP GWAS summary data are publicly available: https://gwas.mrcieu.ac.uk/. ICBP summary data can be applied by contacting Mark Caulfield (m.j.caulfield@qmul.ac.uk) or Paul Elliott (p.elliott@imperial.ac.uk).
